# Supplementary figures and images for: Characterisation of the ABO Blood Group Phenotypes Using Third-Generation Sequencing
Source: Int J Mol Sci. 2025 Jun 6;26(12):5443. doi: 10.3390/ijms26125443 (PMC12192586; doi:10.3390/ijms26125443)

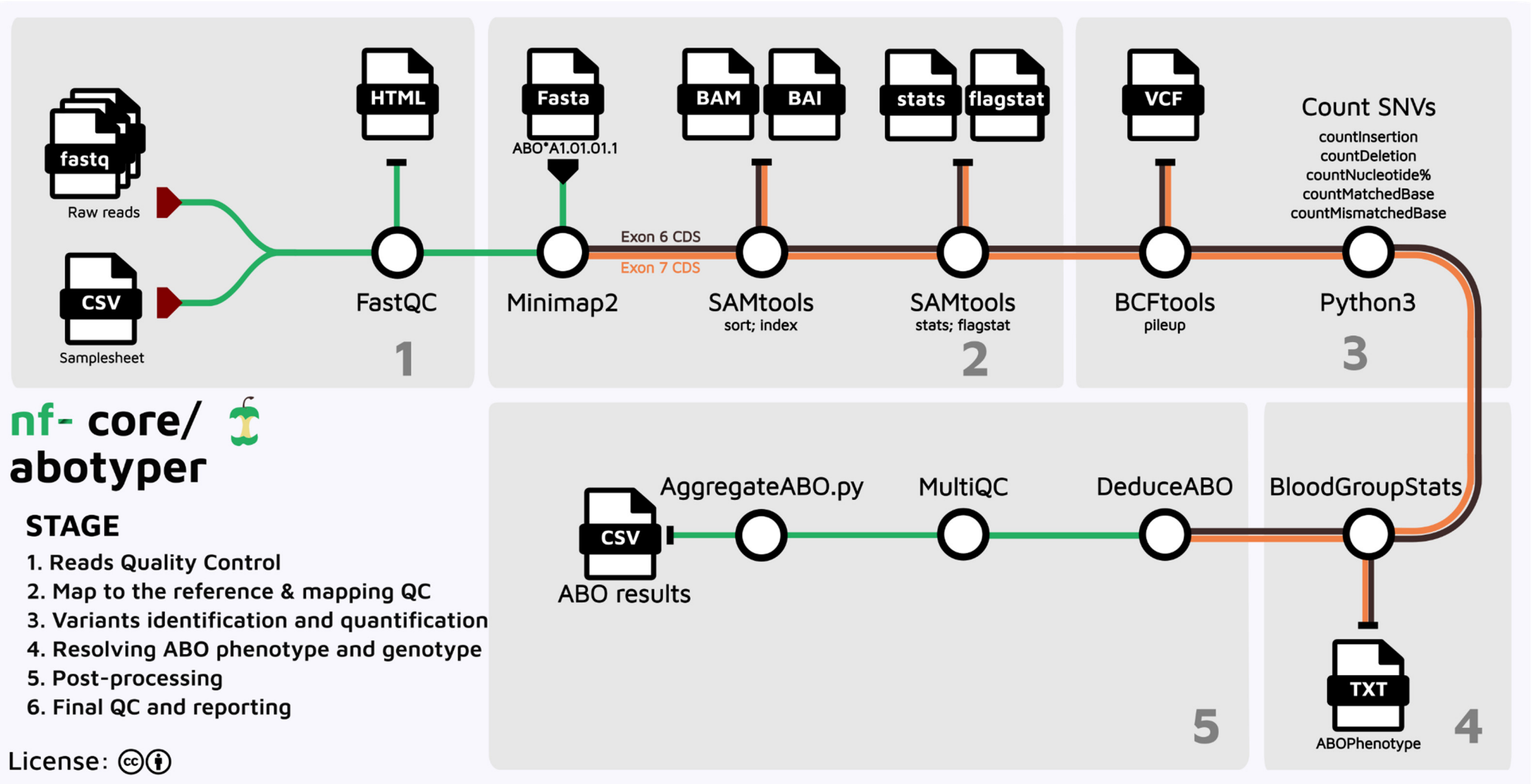

Supplementary Figure S1. A subway map of the ABO blood group phenotype prediction pipeline.

Supplement: Supplementary file 1 [file ijms-26-05443-s001.zip › Figure S1.pdf]
